# Mechanosensitive Pathways in Heart Development: Findings from Chick Embryo Studies

**DOI:** 10.3390/jcdd8040032

**Published:** 2021-03-26

**Authors:** Maha Alser, Samar Shurbaji, Huseyin C. Yalcin

**Affiliations:** 1 Biomedical Research Center, Qatar University, Doha P.O. Box 2713, Qatar; maha.alser@qu.edu.qa (M.A.); ss1104227@qu.edu.qa (S.S.); 2 College of Health and Life Sciences, Hamad Bin Khalifa University, Doha P.O. Box 34110, Qatar

**Keywords:** chick embryo, hemodynamics, mechanobiology, mechanosensitive pathways, surgical interferences, left atrial ligation, outflow tract banding, vitelline vein ligation, chemical treatment, gene expression

## Abstract

The heart is the first organ that starts to function in a developing embryo. It continues to undergo dramatic morphological changes while pumping blood to the rest of the body. Genetic regulation of heart development is partly governed by hemodynamics. Chick embryo is a major animal model that has been used extensively in cardiogenesis research. To reveal mechanosensitive pathways, a variety of surgical interferences and chemical treatments can be applied to the chick embryo to manipulate the blood flow. Such manipulations alter expressions of mechanosensitive genes which may anticipate induction of morphological changes in the developing heart. This paper aims to present different approaches for generating clinically relevant disturbed hemodynamics conditions using this embryonic chick model and to summarize identified mechanosensitive genes using the model, providing insights into embryonic origins of congenital heart defects.

## 1. Introduction

The heart is the first organ to develop in vertebrate’s embryo, and as soon as it forms, it starts to function and remodel simultaneously [1]. Heart development (cardiogenesis) is a conserved process among vertebrates. In animal models—such as the embryonic chick—cardiac development, anatomy, and physiology highly mimic the process in human fetuses. The process involves main steps of neural crest migrations, tube formation, looping, trabeculation, and valve development [2]. Due to this similarity, embryonic chicks serve as a reliable animal model in cardiogenesis and embryology.

Heart development is a very dynamic and sensitive process, and deviations from normal cardiogenesis lead to development of congenital heart defects (CHDs). CHDs vary in severity and consequence, and the source of CHDs’ induction vary from one case to another. Genetic mutations are known to contribute to around 30% of CHDs cases as suggested by epidemiological studies [3], and the origins for the remaining are mainly unknown [4]. Blood flow alterations in the heart at early developmental stages were shown to contribute to CHDs [5,6]. The constant interactions between the force of blood flow through the heart, and developing tissue is called hemodynamics, and the way hemodynamic alterations guide the heart morphogenesis is partially understood. Hemodynamics alterations and their role in inducing CHDs have been extensively studied using animal models, especially chick embryos [7].

The advantages of using embryonic chicks have been reviewed by [8,9]. Briefly, they are easy to access since they develop ex utero. That also gives an advantage to do chemical or surgical manipulations on large numbers of eggs and further study their effect on animal development in situ [10]. Second, the developmental stages of chick embryo have been established since 1951, where Hamburger and Hamilton staged embryonic chicks under 45 stages according to developmental hall marks [11]. That gave researchers the ability to design their studies at consistent time points. Third, as compared to mammalian animal models, embryonic chicks are cost effective. Finally, their genome has been sequenced, and they share 70% of their genome with the human genome (*Homo sapiens*). This high genome similarity led to the conservation of many key mechanisms in metabolism and development [12]. Additionally, in terms of cardiac development, the avian heart resembles human heart anatomy and physiology and has similar developmental stages. Therefore, it is a model relevant to cardiovascular research, while other animal models such as zebrafish are appropriate for genetic or chemical interference [13], and suitable for direct microscopic imaging [14,15]. That is because of simpler heart structure with fewer chambers and valves, these have limited resemblance to human cardiogenesis [8]. Because of that, many studies utilized chick embryo model in cardiac development studies and assessed gene expression levels after inducing cardiac manipulations to study embryonic development of CHDs.

Multiple research groups have reviewed the effect of clinically relevant in vivo microsurgeries or similar interferences on changing cardiac hemodynamics and inducing morphological alterations in heart development resembling human CHDs [6,9,16,17]. Others aimed to synthesize what is known about the genetic pathways for cardiac development and disease [18,19,20,21]. However, up till now, no reviews aimed to link microsurgery or chemically induced hemodynamic alterations and resulting cardiac defects with gene expression alterations. Here, we aim to review the papers that discussed altered hemodynamics (either surgically or chemically) leading to CHD-like defects in embryonic chick model alongside with the available data about the involvement of associated molecular pathways. This synthesized information will highlight important pathways where hemodynamics plays a role and will put the baseline for treatment approaches of different CHDs.

## 2. Avian and Human Heart Development

The heart is a complex organ which forms and starts to function early in a developing embryo. At early stages, it starts beating while constantly remodeling at the same time. Different multipotent progenitor cells that respond to external chemical signals and mechanical cues participate in heart development [22].

Cardiogenesis consists of sequential landmarks that are common between all vertebrates. Mainly, the stages are tube formation, cardiac looping, ventricular trabeculation, and septation/valve development [2]. Cardiogenesis begins at the interior of the primitive embryonic body by the formation of two cardiogenic regions from both sides of the neural plate. Then, these two regions (the cardiac mesodermal primordia) migrate to the ventral midline forming two endocardial hollow tubes, which fuse and form a primitive heart (primitive cardiac loop) [23]. This cardiac tube bends, a process known as looping and spatially develops forming the tubular S shaped heart, which consists of a distinct primitive atrium, a primitive ventricle, and a primitive outflow tract [24]. At this stage, the heart pumps in a peristaltic-like motion, and keeps pumping and developing at the same time. The heart then differentiates into a septated heart with four chambers. Other developmental events include the formation of cardiac cushions (primitive valves), trabeculation, and cardiac remodeling throughout the process. These heart developmental stages are reviewed intensively in many papers, which can be referred to for more details [2,7,25].

The cardiac development events are common between human and chick, and chick heart highly resembles human heart in terms of anatomy, physiology, and development. They both undergo the same developmental steps from primordia fusion to four-chambered heart formation level. Noticeably, there is a remarkable difference in the timings and duration of each step due to the differences in the time scales [2]. In humans, cardiac development begins as early as 18–19 days post fertilization, Carnegie stages are used to define human heart development [26]. In the embryonic chicks, cardiac development usually follows the Hamburger-Hamilton staging system [11].

## 3. Hemodynamics and Congenital Heart Diseases

CHDs are the anomalies that occur in the heart during early embryonic developmental stages, and they are the most common lethal disorders occurring in newborn children [6]. CHDs occur when normal cardiogenesis process gets interrupted, and in some severe cases, heart transplant is a required procedure to save the patient [27]. 

The etiology of CHDs development is multifactorial. Mainly, the interaction between the genetic, molecular, mechanical, and environmental factors plays a role in CHD development. Genetic variations and point mutations explain the minority of the cases [28]; however, 70% of the cases remain unexplained. By observing the heart physiology from a mechanical perspective, hemodynamics come as an important epigenetic factor in CHD development [29]. Hemodynamics, is the dynamics of the blood flow through the heart, and it has been accepted as a major source of CHDs in newborns [30]. This is because the cardiac muscle starts functioning and developing at the same time under certain flow patterns and responds to this flow during cardiogenesis. Thus, developmental biologists aim to study CHDs and how they progress by altering hemodynamics in animal models through different interferences. More specifically, in embryonic chicks, multiple clinically relevant surgical approaches and chemical treatments have been proposed, applied, and investigated to induce hemodynamic alterations and assess the development of specific CHDs. 

The link between hemodynamics and CHDs have been nicely reviewed by Courchaine and colleagues in their paper about the effect of blood flow on cardiac development [17]. Basically, the exact mechanism for how hemodynamic factors govern cardiac development is still unclear. Multiple molecular (including mechanosensing and mechanotransduction) interactions are involved in the process and being studied at the mean time, but further investigations is still required. 

## 4. Mechanosensitive Cardiac Pathways in Embryonic Chick

Cardiogenesis continues under constantly evolving hemodynamic environment [31]. Previous animal studies from our group and others have shown that surgical interference affecting hemodynamics induce clinically relevant cardiac defects [10,19,21,32,33]. 

Altered hemodynamics could lead to cardiac malformations, mainly by altering the gene expression of mechanosensitive pathways that respond to the changes in hemodynamics. This alteration then leads to morphological and developmental changes in the cardiac muscle, causing CHDs. In this section, we will summarize the animal work that have been done on embryonic chicks, where hemodynamics was altered (surgically or chemically) affecting downstream pathways and the expression level of relevant genes.

## 5. Surgical Manipulations

Surgical interventions have been applied for long time on embryonic chick animal model to disturb hemodynamics and induce cardiac defects. As early as 1962, Rychter performed several microsurgeries to induce hemodynamic changes [34]. Inducing altered hemodynamics using surgical method is preferred over genetic and chemical induction in this model. This is because of the size and accessibility offered by the embryonic chick as mentioned in the introduction. Most of the studies performed so far show a relation between the surgery induction and hemodynamics alterations and subsequent developmental changes or defects, which were reviewed thoroughly by [16]. However, not many studies showed the effect of these surgeries on the gene expression level in the vicinity of surgery site exposed to disturbed hemodynamics. Most common surgical approaches on embryonic chick are left atrial ligation (LAL), vitelline vein ligation (VVL), and outflow tract banding (OTB). Some other surgeries were also proposed/applied in few studies, like the right atrial ligation (RAL), which will be reviewed as well.

### 5.1. Left Atrial Ligation (LAL)

**Figure 1:**
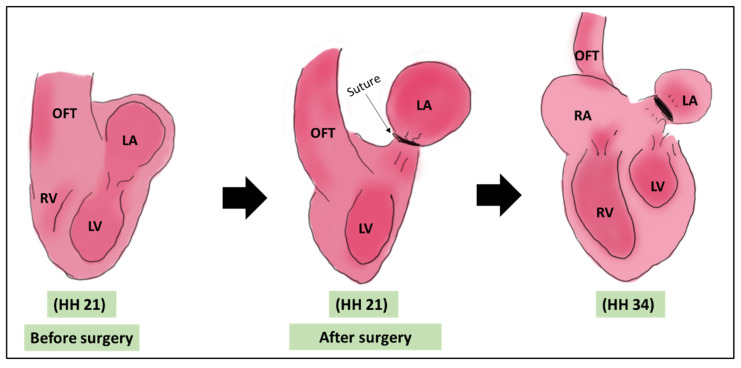
A schematic showing the LAL surgery. The surgery is done on HH 21 where a suture is tightened around the left atrium. The main morphological output of LAL is the underdeveloped LV [5,35]. OFT: outflow tract, LA: left atrium, LV: left ventricle, RA: right atrium, RV: right ventricle, HH: Hamburger & Hamilton.

LAL was first introduced by Rychter and Lemez in 1964 [34]. It is a survival surgery where the left atrium is ligated with a suture to constrict the inflow blood flow. This ligation reduces the atrial volume capacity and decrease the effective volume of the left atrium. This reduction is associated with a subsequent reduction in the size of the left ventricle as it adapts to the intervention [5,35]. The surgery is performed at HH 21 (ED 4 of incubation), where the heart is still not septated (pre-septation stage), (Figure 1). The main defect associated with LAL is left heart hypoplasia (LHH) mimicking human hypoplastic left heart syndrome (HLHS) due to the reduction of blood volume received by the left ventricle from the left atrium [9,36]. The surgery is effective in introducing the HLHS like defect, with 100% of embryos undergone LAL developing hypoplasia at HH34 (ED 8) [37]. 

#### 5.1.1. LAL Induced Hemodynamic Alterations

LAL was shown to induce hemodynamic changes to the developing embryo as it causes an immediate cardiac preload and leads blood to be redirected from the left to the right side of the developing heart. LAL affects basic hemodynamic parameters: it decreases intraventricular pressure, stroke volume, and AV inflow velocities [38]. LAL is a method to induce arterial hemodynamics changes, and it causes an immediate increase in heart rate, a reduction of stroke volume, cardiac output, and hydraulic power at different timepoints post LAL [7]. These changes were associated with an increase in characteristic impedance and a decrease in compliance. According to Tobita & Keller, LAL decreases the maximum AV inflow velocity [35]. Additionally, Tobita and colleagues showed that LAL decreases end-diastolic pressure in operated embryos as compared to control embryos [39]. Kowalski et al., showed that LAL mainly decreases wall shear stress (WSS) in left side of the heart [40]. The same findings were presented recently, where Salman and colleagues conducted CFD analyses on LAL and control hearts and showed that LAL resulted in a decrease in the left AV canal WSS levels at two time points (pre- and post-septation). This reduction was persistent and was compensated by increased WSS in the right side of the hearts [33]. These sustained hemodynamic alterations following the surgery are thought to be the cause of the cardiac structural defects post LAL.

#### 5.1.2. LAL Induced Morphological Alterations

LAL affects the developing hearts at different aspects. Morphologically speaking, LAL leads to more apical rounding, reduced left ventricle myocardial volume, accelerated trabecular compaction in the left ventricle, which is compensated by chamber dilation in the right ventricle of the same heart [16]. Additionally, it was shown that LAL alters ventricular myofiber architecture, and induces hypoplasia in LV chamber, which as well associated with reduced LV cell proliferation rate [41]. More recently, Pesevski et al. showed that LAL induced endocardial fibroelastosis (EFE), revealed from the thickening in the cardiac endocardium in the LAL hearts as a secondary effect of the surgery [42]. As compared to right ventricles, this effect was more pronounced in the left ventricles, where collagen production also increased as a marker of EFE. Additionally, LAL caused morphological changes to the AV valve. Abnormal AV valve was observed as hemodynamic changes interacted differently with the valve and caused underdevelopment of the left AV valve and overdevelopment of the right AV valve [43]. LAL also leads to hypoplastic aortic arch, which highly resembles the clinically observed HLHS [25]. Developmentally, LAL causes reduction in left ventricular vascularization, diminishes its lymphatics, and reduces proliferative cells in the left ventricle. This was compensated by increase in proliferative cell population and enhanced trabeculation in the right ventricle [44]. 

#### 5.1.3. LAL Induced Pathway Alterations

A study was conducted by Sedmera et al on the effect of LAL on the expression levels of cardiac development related genes [44]. They showed that FGF-2 expression was reduced in the left ventricle after LAL, which explains why proliferation was diminished in the left side of LAL hearts. On the other hand, the expression level of FGF-2 main receptor (FGFR) showed a significant increase in both ventricles after LAL. PDGF-α expression significantly increased in the right ventricle trabeculae only, while PDGF-β decreased in all LAL heart region. These findings are summarized in Table 1.

Since LAL is a side specific surgery mainly affecting the left side of the heart, it was reported that it alters hemodynamics in a side-specific manner [25]. Krejčí et al. conducted a study which unraveled differential gene expression between control and LAL hearts at ED8 [45]. The microarray analyses identified 91 genes that were differently expressed between control and LAL hearts, while only 66 were different between right ventricles and left ventricles. A large proportion of the genes (58%) were interpreted as causing delay in maturation, where the expression in LAL was revealed in normal hearts but at an earlier stage. This indicates that hemodynamic alterations lead to developmental delay in a side specific manner, which could be the cause of hypoplasia in the LAL heart. The genes could be found in the supplementary results of their work. From the reviewed papers, it can be concluded that the decreased arterial and AV flow velocities and downregulation of valvulogenesis genes are consistent with underdevelopment of AV cushions, suggesting decreased WSS due to LAL caused this hypoplasia. Table 1 summarizes mechanosensitive pathways revealed from LAL studies.

### 5.2. Vitelline Vein Ligation/Clipping (VVL)

**Figure 2:**
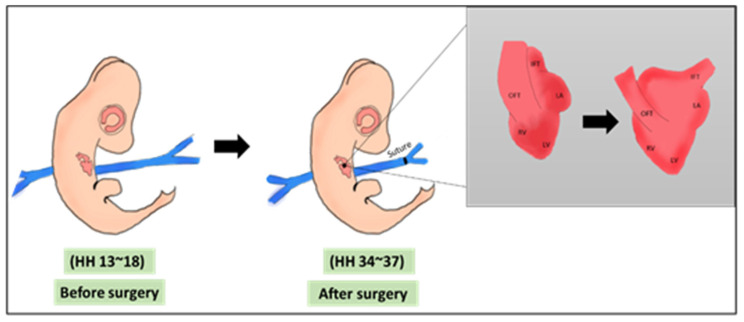
A schematic showing the VVL surgery. The surgery is done on HH 13–18 where a suture is banded around one of the vitelline veins. The main morphological output of VVL is the defected cardiac looping [45]. OFT: outflow tract, IFT: inflow tract, LA: left atrium, RV: right atrium, LV: left ventricle, RV: right ventricle, HH: Hamburger & Hamilton.

The left and right vitelline veins are the main veins that return blood from the yolk sac to the heart. Procedures involving vitelline vein ligation or clipping mainly affect the venous return, a case that mimics reduced placental blood flow [25,46]. VVL, either left or right, induces hemodynamic changes that leads to major alterations in cardiogenesis, especially when performed in early stages. Technically, VVL is performed at HH13–18 (ED 2~3 of incubation) which is an early stage in cardiogenesis, the surgery is shown in Figure 2. At ED2~3, the chick heart is looping and still not septated. The surgery is performed using either ligation or clipping. In ligation, the surgery is performed by tightening a surgical suture around one of the veins, constricting the inflow blood to the heart, while in clipping, a surgical clip is used in the constriction process. It was reported that both methods induce the same hemodynamic alterations to the chick heart [16]. VVL effect on cardiac hemodynamics was thoroughly reviewed in 2014 by Kowalski et al., 2014. Briefly, VVL alters hemodynamic parameters, mainly at AV and OFT valves, cardiac output, heart rate, peak and mean velocity, contractility, and compliance [25]. 

#### 5.2.1. VVL Induced Hemodynamic Alterations

In a study reported by Stekelenburger-de Vos et al. where the right lateral vitelline vein of HH17 embryos were clipped using a microchip, authors obtained eight dorsal aortic blood velocity measurements for each embryo for a period of 5 h. Different hemodynamic parameters were assessed including the heart rate, peak systolic velocity, time-averaged velocity, peak blood flow, mean blood flow, peak acceleration, and stroke volume. The authors reported a significant decrement in all these parameters after the venous clipping. A recovery of three parameters (heart rate, time-averaged velocity, and mean blood flow) during the 5-h period was reported as well. They suggested that the VVL can induce heart malformation by affecting the gene expression of shear responsive genes [47]. Further study was conducted by Rugonyi et.al where they focused on determining hemodynamic changes that occur at the OFT after VVL in HH18 embryos. The authors assessed both cardiac wall motion and blood flow using the optical coherence tomography system (OCT). For OFT wall motion, they found that there is a significant reduction in the ratio between the expansion and contraction time, which suggest a decreased amount of blood flowing through the heart. Furthermore, the authors found that the blood velocity values in VVL embryos is lower than the controls [48]. Midgett et al. conducted a study where hemodynamics of HH18 chicken embryo were altered by right VVL. They reported a decrement in blood flow through the heart and increment in blood pressure and flow velocity after the ligation [6]. As noted, results regarding hemodynamics reported from different studies after VVL are consistent. 

#### 5.2.2. VVL Induced Morphological Alterations

VVL affects hemodynamics and heart development in multiple ways: it changes overall heart shape, and interferes with ventricular septation, and valve development. Since VVL is conducted during heart looping, it was reported to affect the whole heart shape and to increase the distance between inflow tract (IFT) and outflow tract (OFT) causing malformation in the cushion formation [46]. These abnormalities are associated with the symptoms of ventricular septal defects (VSDs). However, different reports showed differences in association of VVL caused defects with VSDs [6]. After VVL is introduced to the right side, 10–72% of VVL embryos developed mild to severe VSDs, while left VVL lead to VSDs in 18% of the embryos. Pharyngeal arch artery malformation (PAA) was also a consequence of VLL where it developed in 30% of right VVL and 9% after left VLL. AV valve malformation was seen in around 8% of right VVL as reviewed by Midgett and colleagues [7].

#### 5.2.3. VVL Induced Pathway Alterations

Few papers linked VVL with mechanosensitive pathways, and these mainly focused on the effect of VVL on the gene expression of shear stress-related genes. Groenendijk et al., 2005 conducted right VVL at HH17 followed by qPCR and in-situ hybridization analyses (1-h post-surgery). They showed that VVL locally affected the expression of shear stress responsive genes. Endothelin-1 expression was decreased, Krüppel-like factor 2 (KLF2) and the endothelial nitric oxide synthase (eNOS) were upregulated. These changes suggest locally increased cardiac shear stress [21]. Another group showed that the shear response alteration induced by VVL at HH17 lead to upregulated gene expression of KLF2 and Activin receptor-like activin receptor 5 (ALK5), which suggests an activation of the transformin growth factor beta (TGFβ)/ALK5 signaling pathway in the cardiac endothelial cells. The effect was reported 3 h post-surgery [49]. Based on that, Espinosa et al. assessed the gene expression of the same genes with additional selected genes using in situ hybridization. They conducted right VVL in HH18 and focused their analyses on the developing aorta (DA) and the extracellular matrix (ECM) proteins. They showed that VVL decreased elastin expression (ELN) and collagen in the ECM. In addition, they reported lysyl oxidase (Lox) upregulation, with no effect on Et-1 expression, and downregulation of KLF2, TGF-β1, and the matrix metallopeptide 9 (MMP9) in the DA [50]. Taken together, VVL induces shear stress in the developing chicks and causes alterations in shear responsive genes in both ECs and DA ECM proteins. The contribution of TGF-β pathway in how VVL affects cardiac development was also confirmed by [51], where it was shown that VVL increased TGF-β receptor III (TBRIII) as a main factor in the TGF-β signaling pathway. These findings are summarized in Table 1.

### 5.3. Outflow Tract Banding (OTB)

**Figure 3:**
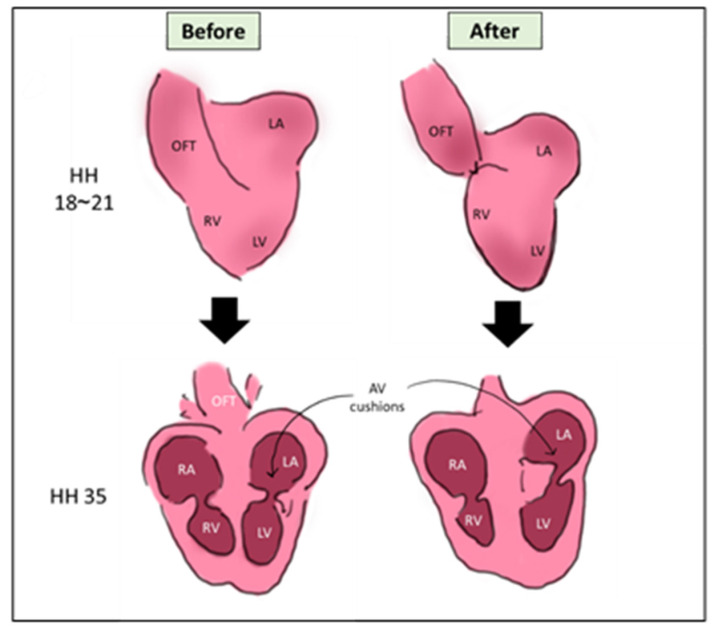
A schematic showing the OTB surgery. The surgery is done on HH18~HH21, where a suture is banded around the OFT. The main morphological output of VVL is the defected looping and cushion development [56]. OFT: outflow tract, LA: left atrium, RA: right atrium, LV: left ventricle, RV: right atrium, AV cushions: atrioventricular cushions, HH: Hamburger & Hamilton.

The outflow tract is the main blood vessel that carries blood leaving the heart to the rest of the body. After septation, it septates into aorta and pulmonary artery. In OTB, a surgical suture is tightened around the outflow tract (OFT) at pre-septation stage, constricting its lumen diameter and limiting the OFT wall movement. Technically, the surgery is performed at HH18-HH21 (ED 3~3.5), where the heart is still in its looping stage as visualized in Figure 3. Similar to the previous surgeries, OTB alters the hemodynamics of the developing heart and leads to cardiac defects changes [9,52].

#### 5.3.1. OTB Induced Hemodynamic Alterations

OTB alters hemodynamics as follows: it first increases the heart pressure within the developing heart due to increased pressure resistance from smaller OFT diameter. It induces a blood flow delay (smaller velocity) from the LV to the OFT, resulting in an increase in wall shear stress in the OFT. Chivukula et al showed the effect of OTB on OFT wall motion as a result of hemodynamic change in that site [30]. They examined the OFT lumen and myocardium expansion movement. As expected, OTB leads to immediate reduction in the maximal lumen area around the suture tightening site. This indicates the effect of OTB on cardiac expansion. Unexpectedly, OTB affected the OFT downstream hemodynamics, away from the banding site, which still partly understood, which might be due to the increased OFT wall stiffness from locally banding the OFT, leading to an increase in wave propagation speed. The same group conducted an OCT analysis on OTB hearts for more OFT motion analysis. They found that OFT lumen closure time during the cardiac cycle increased significantly as compared to control hearts [30].

#### 5.3.2. OTB Induced Morphological Alterations

The major effect of OTB is the immediate increase in cardiac afterload, which alters the normal cardiac looping, septation, and cushion development. Different reports have shown different outcomes of OTB. OTB development associated malformations included accelerating ventricular trabecular remodeling and compaction [5], pharyngeal arch artery malformations (PAA) [53], increased ventricular stiffness [54], and abnormal valvulogenesis in the AV valve [5], and the OFT valve [55]. Septation defects included ventricular septal defects (VSD) and double outlet right ventricle (DORV) [5]. A group induced OTB at HH 21 and harvested the hearts at late cardiac development stages (HH 26, 29, and 35). Their assessment that OTB affected the size of the valves in a similar way it affected the size of the cushions at earlier stages, including smaller leaflets, thickening of the leaflet ends, and VSD development [56].

#### 5.3.3. OTB Induced Pathway Alterations

Menon et al. studied the effect of OTB on OFT valvulogenesis. Their gene expression assessment involved OFT valve crucial genes. rhoA, collagen1α1, and CDH11 were significantly downregulated after OTB, while Periostin, TGF-βRIII, TGF-β3, and MMP2 levels were significantly upregulated. mRNA expression of MMP2 was significantly upregulated. The expression of Elastin, Vinculin, TGF-β2, and Filamin were not significantly different between OTB and control hearts [55]. The same group recently continued studying the surgery and assessed the effect of reversing it on the gene expression level of most of the genes mentioned earlier. Menon et al. conducted the surgery, rescued the hearts, and allowed them to recover for 24 and 48 h. Post recovery, they assessed the gene expression level of genes of interest. The transcription level of TGF-β ßRIII and CDH11 was recovered to control level after rescue, which indicates that the OTB hemodynamic change is reversible in terms of gene expression. On the other hand, the expression of Periostin and TGF-β3 remained altered after the two time points of recovery [57]. 

Pang et al., 2017 were mainly interested in the effect of OTB on valvulogenesis, and thus they focused their gene expression assessment on valve development markers. They reported a significant reduction in TBX20, a major transcription factor in valvulogenesis, over the whole heart. Aggrecan, a downstream factor of TBX20 was not significantly reduced [56]. Shear-responsive genes were also assessed, KLF1 showed a significant increase in OTB hearts, while EDN1 was downregulated in both ventricles. ECM related genes were tested to show the effect of OTB on ECM development. Fibrillin 2 (FBN2), tenascin C (TNC), collagen III A1 (COL3A1), and transglutaminase 2 (TGM2) were significantly downregulated after OTB [56]. Based on the ECM genes findings, Perdios et al. assessed the expression of DDR2 and collagen12A1 (COL12A1) post OTB. Their qPCR analysis showed overexpression of both genes in the OTB hearts [52]. An interesting paper showed the work done by Celik and colleagues, where they studied the effect of OTB and reversing OTB on the expression level of matrix structure genes as well, but focused on the aortic arch part of the heart. They showed that OTB decrease the expression of collagen I (COL-I) Elastin (ELN), and VEGF in the aortic arch. The expression level of these factors was restored when OTB was reversed [58]. These findings are summarized in Table 1.

To sum up the effect of OTB on cardiac development, it was shown that OTB changes hemodynamics by altering the heart pressure, wall shear stress in the OFT and ultimately cardiac afterload. These changes lead to morphological defects mainly in the OFT valve, looping, and septation. The changes also lead to changes in the gene expression of valvulogenesis important genes and ECM related genes, in some genes the effect was reversible, and the normal expression level was restored after rescuing the heart. These findings support the critical importance of the cardiac afterload and hemodynamics for valvulogenesis, ECM building, and heart development in general.

### 5.4. Right Atrial Ligation (RAL)

Based on the observed results seen in LAL, and the side-specific effect of the surgery on the hearts, right atrial ligation (RAL) was proposed by our group as a hemodynamic altering surgery in the heart [43]. Similar to LAL, the surgery is performed by tightening a surgical knot around the right atrium at HH 21 (ED 4), constricting the blood flowing from right atrium to the right side of the common ventricle. Based on the data observed in the LAL section (1.1), RAL is thought to have an opposite effect in terms of heart side development. Limited data is published yet to show the outcomes hemodynamic alterations. Gould et al. applied RAL to show that hemodynamic alterations due to RAL resulted in underdevelopment of right AV valve at HH21 (ED 3.5), post septation stage, suggesting RAL decreases flow velocity in right side of the heart [43]. Preliminary data observed by our group suggests that the surgery does not induce drastic morphological defects similar to LAL. This may be either due to lower plasticity of right ventricle compared to left ventricle or lower hemodynamic alterations due to RAL compared to LAL. Ongoing research is being conducted to establish RAL as a surgical procedure on chick embryos, and to study its downstream effects.

**Table 1:** Summary of surgical interventions and the genes they up/downregulate in the embryonic chick specific heart regions.

Study	Interference	Gene	Expression Level	Surgery HH Stage	Assessment HH Stage	Region
[44]	Surgical (LAL)	FGF2	down	21	29 and 34	LV
		FGFR	up	21	29 and 34	LV, RV
		PDGF-α-β	up	21	29 and 34	RV
		PDGF-β	down	21	29 and 34	whole heart
[45]		91 genes	variable	21	34	LV, RV
[21]	Surgical (VVL)	ET-1	down	17	17	Sinus vinosus
		KLF2	up	17	17	endocardium
		eNOS	up	17	17	OFT cushion endocardium
[49]		ALK5	up	17	17	Endocardial cushions
[50]		ELN	up	18	36	ECM
		collagen	up	18	36	ECM
		Lox	up	18	36	DA
		ET-1	no effect	18	28, 33, 36	DA
		KLF2	down	18	34	DA
		TGF-β1	down	18	34	DA
		MMP9	down	18	34	DA
[51]		TBR III	up	17	35	LA and RA endocardium and ventricular trabeculations
[55]	Surgical (OTB)	rhoA	down	17	19	OFT valve
		collagen1a1	down	17	19	OFT valve
		CDH11	down	17	19	OFT valve
		Periostin	up	17	19	OFT valve
		TGF-β RIII	up	17	19	OFT valve
		TGF-β3	up	17	19	OFT valve
		MMP2	up	17	19	OFT valve
		ELN	no effect	17	19	OFT valve
		Vinculin	no effect	17	19	OFT valve
		TGF-β2	no effect	17	19	OFT valve
		Filamin	no effect	17	19	OFT valve
[56]		TBX20	down	21	35	whole heart
		Aggrecan	no effect	21	35	whole heart
		KLF2	up	21	29	whole heart
		EDN1	down	21	35	LV and RV
		FBN2	down	21	35	whole heart
		TNC	down	21	35	whole heart
		COL3A1	down	21	35	whole heart
		TGM2	down	21	35	whole heart
[52]		DDR2	up	21	35	whole heart
		COL12A1	up	21	35	whole heart
[58]		COL-1	down	18	24	aortic arch
		ELN	down	18	24	aortic arch
		VEGF	down	18	24	aortic arch

## 6. Chemical Manipulations

Altering hemodynamics using chemical treatment is notably less common as compared to surgical methods, mainly because chemical treatments might have more off targets other than altering the blood flow in the heart. Few papers used different chemicals in order to alter hemodynamics and studied the effect of these treatments on the gene expression of selected genes, which is reviewed in this section.

### 6.1. Trichloroethylene (TCE)

TCE is a synthetic solvent that is commonly used in industry, it is a volatile agent that could be found in the environment as a contaminant [59]. TCE’s maximum contaminant level is defined as 5 ppb in water [60]. TCE affects cardiac development by misregulating Ca^2+^ homeostasis in cardiomyocytes, but also by influencing hemodynamics alterations. Few groups studied the effect of embryonic chick exposure to TCE in terms of hemodynamics and cardiac development-related genes. The first group to show that TCE is a cardioteratogen and linked its effect on hemodynamics was Drake et al., where they performed Doppler ultrasound analysis post TCE treatment and showed reduction in blood flow in both AV and OFT valves after exposure to 8 ppb of TCE [61]. 

Based on these findings, Makwana and colleagues studied the effect of different TCE doses on embryonic check and assessed cardiac functions and gene expression. They reported that TCE had a negative impact on the cardiac function as it increases the half width of cardiomyocyte contractility, leading to CHD progression. The effect of TCE was also seen at the level of gene expression of shear responsive genes, which also plays a role in CHD formation. A qPCR analysis unraveled that TCE treatment led to significant reduction of mRNA levels of both eNOS and KLF2 markers after 8pbb TCE treatment, while the levels of ET-1 where variable [62]. 

Based on the association between TCE and cardiac defects, the group continued to work on TCE and gene expression. In 2013, Makwana et al treated embryonic chicks with 8 ppb of TCE and conducted qPCR analysis, which showed an overexpression of CYP2H1 mRNA in the cardiac tissue. The observation was confirmed at the protein level by immunostaining of CYP2C in the myocardium and endothelium. These genes encode for detoxification enzymes, and their response to TCE is explained by the fact that TCE is toxic in its nature [63]. In 2018, TCE was shown to inhibit the expression of the hepatocyte nuclear factor 4 alpha (HNF4-α), which is known as a toxicity responsive gene with no previous knowledge about its role in the heart [60]. Combined, these findings show that TCE affects embryonic cardiac health by altering hemodynamics, inducing the expression of shear responsive genes as well as toxicity responsive genes. These findings are summarized in Table 2.

### 6.2. Homocysteine (HCY)

HCY is a non-essential amino acid, but a precursor of different amino acids, which are highly demanded at early development embryonic stages. High HCY in the blood stream indicates that it is not consumed in the form needed by the fetus. Thus, hyperhomocysteinemia (high homocysteine in the blood) was found to be associated with the development of CHDs [64], especially in the OFT part of the heart [65]. It was also confirmed that HCY induces CHDs in the chick embryo by inhibiting the conversion of retinal to retinoic acid, which is critical at early developmental stages [64]. Oosterbaan et al. was the first to link homocysteine to hemodynamics in the embryonic chicks. They conducted doppler analysis at ED 3.5 (pre-septation) and showed a significant reduction in heart rate and shift in hemodynamic parameters and wave times (a wave and ejection wave). These observations are comparable effect of surgical treatment like VVL [66].

Rosenquist and colleagues reported for the first time the effect of homocysteine on the transcription of selected genes. They conducted microarray analysis and succeeded to identify 65 altered genes. These genes belong to families. Some of the genes play roles in cell migration and adhesion (like DAPLE, MYH10, and SEMA6D). Others play roles in metabolism (like the ARFGEF1, ARL13B, SH3KBP1, and PAR-3), DNA/RNA interaction (TTF1, RINZF, RAD50, and RIF1), cell cycle (RRM2B, ASK, ATRX, and DUSP), and transporter/receptor proteins encoding transcripts (STAU, TRPC7, IPO9, and IPO7) [67].

Another group have also shown the effect of HCY on the expression of selected genes. They showed that HCE lead to reduction of VEGFa and its receptor VEGFR-2. This reduction led to morphological changes in the embryonic chick hearts, primarily by reducing the extra embryonic vascularization, change in vascular beds, which are controlled by the presence of sufficient VEGFa and VEGFR-2 [68]. All of these findings are summarized in Table 2.

### 6.3. Thalidomide

Thalidomide is a synthetic drug that have been used to treat different cancers. Recently, it was proven to be unsafe to be consumed by pregnant women is it may lead to CHDs in the fetus. The main effects of thalidomide are CHDs during early cardiogenesis, limb deformities, neural abnormalities, cardiac looping, and lump formation in the heart [69]. To assess the effect of thalidomide on the gene expression, they exposed the embryonic chicks to the chemical and isolated the lumps formed in the cardiac tissues. Their analysis showed significant reduction in immune-related genes IL-6, IFN-γ, TLR1, TLR3, TLR4, and TLR7. No studies showed the effect of thalidomide on the gene expression of cardiac development related markers [69]. These findings are summarized in Table 2.

**Table 2:** Summary of chemical interventions and the genes they up/down-regulate in the embryonic chick specific heart regions.

Study	Interference	Gene	Treatment HH Stage	Assessment HH Stage	Expression Level	Region
[62]	Chemical (TCE)	eNOS	13	17–24	down	Whole heart
		KLF2	13	17–24	down	Whole heart
		ET-1	13	17–24	variable	Whole heart
[63]		CYP2H1	13	17	up	Myocardium, endothelium
[60]		HNF4-α	17	20−23	down	Whole heart
[66]	Chemical (HCY)	VEGF-α	7	18	down	Whole heart
		VEGFR2	7	18	down	Whole heart
[67]		DAPLE	9	12	down	Neural crest
		MYH10	9	12	down	Neural crest
		SEMA6D	9	12	down	Neural crest
		ARFGEF1	9	12	down	Neural crest
		ARL13B	9	12	down	Neural crest
		SH3KBP1	9	12	down	Neural crest
		PAR-3	9	12	down	Neural crest
		TTF1	9	12	down	Neural crest
		RIF1	9	12	down	Neural crest
		RINZF	9	12	down	Neural crest
		RAD50	9	12	down	Neural crest
		RRM2B	9	12	down	Neural crest
		ASK	9	12	down	Neural crest
		ATRX	9	12	down	Neural crest
		DUSP	9	12	down	Neural crest
		STAU	9	12	down	Neural crest
		TRPC7	9	12	down	Neural crest
		IPO9	9	12	down	Neural crest
		IPO7	9	12	down	Neural crest
[69]	Chemical (thalidomide)	IL-6	8	37	down	Whole heart
		IFN-γ	8	37	down	Whole heart
		TLR1	8	37	down	Whole heart
		TLR3	8	37	down	Whole heart
		TLR4	8	37	down	Whole heart
		TLR7	8	37	down	Whole heart

## 7. Conclusions

In conclusion, hemodynamics alterations play a major role in cardiac development and morphogenesis. Embryonic chick is a powerful model to study this relation. Inducing hemodynamic alterations for embryonic chick can be achieved either surgically or chemically. These alterations were shown to induce over expression or under expression of genes important in valvulogenesis, trabeculation and cell proliferation, ECM synthesis, cardiac looping, and aorta development. These stages are important in cardiogenesis, and thus altering these pathways leads to morphological changes and the induction of de novo CHDs. Studying these pathways puts the basic understanding of CHD biology and pathology, and how can they be reversed. 
